# Disembodied AI and the limits to machine understanding of students' embodied interactions

**DOI:** 10.3389/frai.2023.1148227

**Published:** 2023-03-03

**Authors:** Mitchell J. Nathan

**Affiliations:** MAGIC Lab, Wisconsin Center for Education Research, Educational Psychology Department, School of Education at the University of Wisconsin–Madison, Madison, WI, United States

**Keywords:** artificial intelligence, augmented intelligence, cognitive science, embodied learning, foundation models, learning sciences, multimodality

## Abstract

The *embodiment turn* in the Learning Sciences has fueled growth of multimodal learning analytics to understand embodied interactions and make consequential educational decisions about students more rapidly, more accurately, and more personalized than ever before. Managing demands of complexity and speed is leading to growing reliance by education systems on disembodied artificial intelligence (dAI) programs, which, ironically, are inherently incapable of interpreting students' embodied interactions. This is fueling a potential *crisis of complexity*. *Augmented intelligence* systems offer promising avenues for managing this crisis by integrating the strengths of omnipresent dAI to detect complex patterns of student behavior from multimodal datastreams, with the strengths of humans to meaningfully interpret embodied interactions in service of consequential decision making to achieve a balance between complexity, interpretability, and accountability for allocating education resources to children.

## 1. Introduction

The primary objective of this *Perspectives* article is to expose a looming crisis of complexity: educational systems are becoming more dependent on artificial intelligence (AI) programs to make consequential decisions about learning and learners from rich streams of multimodal data that emerge from many sources, including students' embodied interactions. However, disembodied AI (dAI) programs–I argue–are fundamentally incapable of understanding people's embodied interactions in the ways that humans understand them. Furthermore, the emergent dAI models are of such complexity that end users (and often the original programmers) cannot understand the models or recreate the chain of reasoning that led to these decisions. Therefore, dAIs should not be directing consequential educational decisions affecting the lives of children. The secondary objective is to offer potential paths forward from this crisis. One promising approach is the development of “augmented intelligence” systems (AISs) that amplify human performance using dAI resources while relying ultimately on human decision making.

## 2. Theoretical framework: The embodied turn and growth of multimodal learning analytics

### 2.1. The embodied turn in the learning sciences and education

Empirical evidence and arguments from philosophy, psychology, neuroscience, education, and critical theorists in education effectively dismantle the view of learning as information processing of ungrounded symbol systems by dAI that are amodal (i.e., non-sensorial), arbitrary (i.e., non-historical and non-cultural), and abstract (i.e., ungrounded) (Harnad, 1990; Varela et al., 1991; Glenberg, 1997; Shapiro, 2019). To the contrary, humans make meaning of events, ideas, and cultural and scientific inscriptions by grounding them to their sensorimotor experiences that are interpreted within sociocultural and historical contexts (Wilson, 2002; Barsalou, 2008; Newen et al., 2018).

In psychology, Glenberg and Robertson (2000) found that human readers judge the sensibility of sentences based on the sensorimotor affordances invoked by the actions described in the sentences, rather than their lexical interconnections in high-dimensional spaces, as modeled by dAI systems widely applied in education areas such as automated essay grading (LSA; Burgess and Lund, 1997; Landauer and Dumais, 1997).

Neural imaging data show that reading words with motor associations—such as kick, lick, and pick—selectively activates the motor areas of the brain for one's feet, tongue, and fingers, respectively (Pulvermüller, 2005). Botox patients whose injections temporarily paralyze the facial corrugator supercilli muscle used in frowning showed selective impairment in processing sentences that invoke anger but not those that invoked joy or were emotionally neutral (Havas et al., 2010).

Critical theorists in education reject the disembodied view that neglects the central role of culture in language, thinking, symbols, and emotion for educational attainment. McKinney de Royston et al. (2020) expressly identify the essential nature of embodied cultural experiences by framing learning as rooted in bodies and brains that are embedded in social and cultural practices and shaped by lifelong culturally organized activities.

Drawing on these critiques, some education scholars conclude that the knowledge and educational practices of students and teachers are fundamentally determined by people's individual and collective embodied processes in order to make sense of their school-based learning experiences (e.g., Shapiro and Stolz, 2019; Nathan, 2021; Macrine and Fugate, 2022). This has led to innovative designs in embodied learning through educational technology (Papert, 2020; Abrahamson and Lindgren, 2022), embodiment in AI and education (Timms, 2016) and embodied conversational agents (Cassell, 2001) that promote student learning and intellectual development.

### 2.2. Growth of multimodal learning analytics

With the embodiment turn has emerged methods for collecting and analyzing *multimodal data* to model embodied interactions (Worsley and Blikstein, 2018; Abrahamson et al., 2021). These include data for analyzing gestures (Closser et al., 2021), eye gaze (Schneider and Pea, 2013; Shvarts and Abrahamson, 2019), facial expression (Monkaresi et al., 2016; Sinha, 2021), grip intensity (Laukkonen et al., 2021), and so on, coupled with traditional statistical methods, qualitative methods, and deep learning algorithms that model human behavior based on massive amounts of mouse click and text-based data (e.g., Facebook's DeepText, Google's RankBrain). This shift in research methods has been enabled by the proliferation of low-cost, high-bandwidth cameras and sensors that track biometrics, facial, and body movement that supplement field notes, speech, text chat, and click log data (Schneider and Radu, 2022).

Work with multimodal data has historically been labor-intensive and subject to the severely limited processing capacities of humans that constrain the amount of data under consideration, its dimensionality, and the cycle time between data collection, interpretation, and action. This restricted the ability to use multimodal data to identify latent patterns and inform practitioners in real time about embodied interactions relevant to on-task and off-task behavior. Some of the forces that propelled educational data mining and learning analytics (Aldowah et al., 2019; Baker and Siemens, 2022) have motivated the creation of more efficient data analytic tools and algorithms to process massive multimodal corpora (e.g., An et al., 2019; Järvelä et al., 2019). This is leading to the emergence of new methodological practices of *multimodal learning analytics and data mining* (hereafter MMLA; Blikstein and Worsley, 2016).

## 3. Analytic method and evidence: The disconnect between dAI and human meaning making

An analysis of the computational architectures of classical and contemporary AI systems that underly the tools for MMLA reveals that they are fundamentally incapable of understanding the meaning of people's embodied interactions, even as they give the appearance of mimicking intelligent embodied behavior.

Classical, symbol-based AI systems were designed and implemented by human programmers to emulate human intelligence. The arbitrary, amodal, and abstract nature of these symbol systems was a feature, not a bug, and key to the power of these computational algorithms to operate consistently and efficiently, across a wide range of domains. For example, semantic nets presumably could model any organization of memory (Collins and Loftus, 1975). Although classical AI systems excelled at the analytic tasks that are the signature of adult intellect, such as complicated calculations and hierarchical inference-making, they were wholly inadequate at performing culturally familiar tasks well within reach of children, such as balance, face recognition, and basic social interactions (e.g., Resnick, 1987) and struggled to be adaptive in the face of task, environmental, and user variation.

Connectionist architectures arose that addressed many limitations of classical AI. Often, these drew on parallel and distributed forms of computation that adapted to training experiences through the adjustment of strengths of connections among simple nodes in large networks, mediated by hidden layers (McClelland et al., 1986; Rumelhart et al., 1988). These systems excelled at simple pattern learning and prediction, and at many of the sensorimotor skills that eluded early symbolic AI systems. Yet these connectionist systems found many symbol analytic tasks cumbersome. These systems depended heavily on carefully cultivated training sets and pre-coded sensory inputs for successful learning, underscoring their disembodied nature.

New approaches arose that exploited high-dimensional spaces for computing variability and similarity, greatly expanding the training sets they could accommodate and the complexity of the associations they could encode (e.g., Burgess and Lund, 1997; Landauer and Dumais, 1997). Thus, attention in AI development turned to the importance of training experiences and the sheer number of nodes and inter-nodal connections used by these systems.

This fueled the current movement to Foundation AI systems such as BERT, GPT-3, and DALL-E that are built to accommodate enormous training corpora with massive numbers of internodal connections (Bommasani et al., 2021). Foundation AI systems are designed to learn on their own and be adaptive to completely new, untrained conditions—often in ways that their creators cannot foresee. For example, GPT-3 is built on 175 billion parameters trained on 570 Gigabytes of text. GPT-3 can learn to write original essays, produce computer code, and generate reasonable responses to novel discourse (not just novel syntactic structures) it has never been trained on.

Still, these systems are working from disembodied patterns extracted from the regularities of how words and images occur in the training datastreams. GPT-3, as a representative example, “lacks intentions, goals, and the ability to understand cause and effect” [Percy Liang, Director of Stanford's Center for Research on Foundation Models (CRFM), in CRFM, 2021] that naturally come from human being's embodied interactions with one's environment and other people. Newer language models, such as ChatGPT, are based on GPT-3 architecture and develop their language generation and comprehension capabilities through these same basic analytic methods, coupled with a mechanism of Reinforcement Learning from Human Feedback (RLHF; Ouyang et al., 2022) from human labelers. Despite its fascination in the media, RLHF has significant limitations as noted by the developers (Ouyang et al., 2022). Its future performance is based on a number of subjective and untested sources of human bias; specifically: unaccounted for biases of the human labelers and the researchers who initially developed the instructions used by the labelers; the prompts provided by the developers and early users; and that the same human biases are present in the training and model evaluation process. Furthermore, foundation models like GPT-3, ChatGPT, and the like are completely opaque: the creators do not know how the models will work in new domains and cannot predict the future interactions of their creations What's more, in what is both a profound strength and a serious weakness, architectural and training decisions made early on influence a system throughout its lifetime. Thus, when key considerations such as embodiment are neglected, one cannot simply go back and retrofit changes (Bommasani et al., 2021).

These issues of disembodiment, opaqueness, and developmental fixedness all converge to shape a distorted image of what the educational community should be drawn to. As Liang notes in a recent webinar (CRFM, 2021), ideally, “the ethical and social awareness needs to be integrated into the technological development.” However, the norm for social and ethical considerations is to follow *after* the technology is built, trained, and deployed. Liang laments “At that point I think it's too late [Because of emergence and homogenization] some of the critical decisions have been made already, in a structural way” (CRFM, 2021).

Despite their enormous computing power, dAI programs for MMLA are fundamentally incapable of deriving human-centered meaning from embodied interactions. dAI programs fail along philosophical grounds to achieve intentionality (Searle, 1980). Instead, they generate ungrounded models of behavior linked to high-dimensional statistical regularities of behavior, rather than the meaningful embodied experiences they purport to model (Harnad, 1990). They fall short phenomenologically by relying on mathematical redescriptions that intervene between sensation and action (Gallagher, 2018). And the symbol structures they generate to describe human behavior have no cultural or historical bases (McKinney de Royston et al., 2020). As Barsalou (1999, p. 608) states, “computers should not be capable of implementing a human conceptual system, because they do not have the requisite sensory-motor systems for representing human concepts.”

## 4. Urgency of the problem of dAI in educational decision making

A variety of automated detectors have been developed that use non-invasive methods to classify students' emotional states, engagement, and cognitive presence during their participation in on-line classes (e.g., Baker et al., 2010; Liu et al., 2019, 2023).

The increasing availability of multimodal data has coincided with growing expectations for computers to deliver data-driven, real-time directives for education, such as personalized learning (Walkington, 2013) and assessment, added pressures from a global pandemic that disrupted standard, in-person learning, and a lack of oversight or regulation on the access and use of such data by machines in educational settings (Crawford, 2021). The response has been a proliferation of dAI-based solutions to traditional educational problems such as formative and summative assessment and differentiated curricula using tools, such as *4 Little Trees*, that uses eye gaze, facial expression, and body movement to make educational decisions and evaluations about student attentiveness and level of engagement (Chan, 2021; Harper et al., 2022); and systems such as TalkMoves, that collect recordings of classroom discourse but ignore students' non-verbal interactions (Suresh et al., 2021).

The urgency is that school leaders and classroom teachers looking to manage their workloads with limited resources see dAI-based systems as ready-made solutions (e.g., Tyson, 2020). However, school leaders and teachers may be ill-informed about the actual inner workings of dAI systems and the inherent limitations of these systems to understanding people's embodied interactions in the ways that humans understand them, as described in section 2. This needs to change before educational practices become too dependent on dAI systems without proper considerations of ways to address these limitations (as outlined in the next section).

The potential risks are that students' embodied ways of expressing their reasoning are disregarded, thus providing impoverished accounts of their engagement and learning; or, that these non-verbal behaviors are incorrectly classified due to the limitations and biases built into the dAI systems. In both scenarios, dAI systems would be given authority over consequential decisions about students' educational experiences that can have lifelong consequences without adequate oversight by educators.

## 5. Pathways forward

Given dAI limitations, alternatives are needed to manage the complexities of embodied interactions while still offering time-sensitive, human-centered interpretations and accountable decision-making. The emergence of augmented intelligence systems (AISs; Dubova et al., 2022) in areas such as healthcare with high-levels of personal interactions (Crigger et al., 2022) and need for trust ([HLEG-AI] High-Level Expert Group on Artificial Intelligence, 2019) offer promising avenues for education. One exemplar is *detector-driven interviewing* (DDI) methods. DDIs use dAIs to continually monitor human behavior using non-invasive methods for cognitive and affective patterns that signal learning and engagement events of importance to educators (e.g., frustration detectors), then alert human researchers and practitioners of these events to trigger personalized attention, natural human interactions, and customized pedagogical support (Baker et al., 2021; Ocumpaugh et al., 2021; Hutt et al., 2022). Successful DDIs in the learning system *Betty's Brain* (Leelawong and Biswas, 2008) demonstrates its ability to improve educational responsiveness that enhances student engagement and contributes to scientific models of the cognitive and affective processes that shape learning.

## 6. Discussion

The *embodiment turn* in the Learning Sciences dismantles accounts of intellectual behavior that equates cognition with disembodied computation. The rise of MMLA applied to student education is fueling a quiet movement to accede human educational decision making to dAI systems. This essay uses an embodiment framework to argue that autonomous dAI systems are fundamentally incapable of understanding embodied interactions the ways that humans understand embodied interactions due to their disconnect from sensorimotor and sociocultural interactions with their environments, and therefore should not be directing consequential educational decisions. Thus, there is a looming crisis of complexity as dAI systems fundamentally incapable of understanding embodied interactions will be enlisted to manage the enormous complexities of the multimodal models used to describe those embodied interactions and make consequential educational decisions for students. Ethical and embodied AI systems seem a long way off. The time is ripe to invest in alternatives such as augmented intelligence systems that cultivate the omnipresence and computational power of dAIs with the embodied meaning making of human interpreters and decision makers (as illustrated by approaches such as detector-driven interviewing) as a means to achieve an appropriate balance between complexity, interpretability, and accountability for allocating education resources to our children.

## Data availability statement

The original contributions presented in the study are included in the article/supplementary material, further inquiries can be directed to the corresponding author.

## Author contributions

The author confirms being the sole contributor of this work and has approved it for publication.
